# Antifungal susceptibility and in vitro virulence characteristics of clinical *Magnusiomyces/Saprochaete* isolates: a multicenter study from Türkiye

**DOI:** 10.1038/s41598-026-42967-1

**Published:** 2026-03-15

**Authors:** Ali Ozturk, Merve Aydin, Elif Ayca Sahin, Dolunay Gulmez, Elif Seren Tanrıverdi, Baris Otlu, Sevtap Arikan-Akdagli, Bashar Ibrahim, Betil Ozhak, Ozlem Koyuncu Ozyurt, Yasemin Oz, Sebahat Aksaray, Deniz Turan, Muge Aslan, Ayse Baris, Ayse Nedret Koc, Gonca Erkose Genc, Zayre Erturan, Beyza Ener, Nazmiye Ulku Tuzemen, Ebru Evren, Zeynep Ceren Karahan, Aydin Karaarslan, Melda Ozdamar, Berna Gultekin Korkmazgil, Halil Er, Ozgul Cetinkaya, Zeynep Arzu Ilki, Elvan Sayin, Asuman Birinci, Esra Ozkaya, Ilknur Tosun, Rabiye Altinbas, Emine Kucukates, Ayse Kalkanci

**Affiliations:** 1 Department of Medical Microbiology, Faculty of Medicine, Nigde Omer Halisdemir University, Nigde, Turkey; 2 Department of Medical Microbiology, Faculty of Medicine, Erzincan Binali Yildirim University, Erzincan, Turkey; 3 Department of Medical Microbiology, Faculty of Medicine, Gazi University, Ankara, Turkey; 4 Department of Medical Microbiology, Faculty of Medicine, Hacettepe University, Ankara, Turkey; 5 Department of Medical Microbiology, Faculty of Medicine, Inonu University, Malatya, Turkey; 6 Department of Pharmaceutical Microbiology, Faculty of Pharmacy, Suleyman Demirel University, Isparta, Turkey; 7 Department of Medical Microbiology, Faculty of Medicine, İzmir Bakırçay University, İzmir, Turkey; 8 Department of Medical Microbiology, Faculty of Medicine, Akdeniz University, Antalya, Turkey; 9 Department of Medical Microbiology, Faculty of Medicine, Eskişehir Osmangazi University, Eskisehir, Turkey; 10 Department of Medical Microbiology, Haydarpaşa Numune Training and Research Hospital, Health Sciences University, Istanbul, Turkey; 11 Department of Medical Microbiology, Etlik City Hospital, Ankara, Turkey; 12 Department of Medical Microbiology and Medical Mycology, Sisli Hamidiye Etfal Training and Research Hospital, Health Sciences University, Istanbul, Turkey; 13 Department of Medical Microbiology, Faculty of Medicine, Erciyes University, Kayseri, Turkey; 14 Department of Medical Microbiology, Istanbul Faculty of Medicine, Istanbul University, Istanbul, Turkey; 15 Department of Medical Microbiology, Faculty of Medicine, Bursa Uludag University, Bursa, Turkey; 16 Department of Medical Microbiology, Faculty of Medicine, Ankara University, Ankara, Turkey; 17 Department of Clinical Microbiology, Anadolu Medical Center, Izmit, Turkey; 18 Department of Medical Microbiology, Faculty of Medicine, Aydin Adnan Menderes University, Aydin, Turkey; 19 Department of Medical Microbiology, Antalya Training and Research Hospital, Antalya, Turkey; 20 Department of Medical Microbiology, Faculty of Medicine, Marmara University, Istanbul, Turkey; 21 Department of Medical Microbiology, Faculty of Medicine, Ondokuz Mayıs University, Samsun, Turkey; 22 Department of Medical Microbiology, Faculty of Medicine, Karadeniz Technical University, Trabzon, Turkey; 23 Department of Medical Microbiology, Izmir City Hospital, Izmir, Turkey; 24 Department of Medical Microbiology, Cerrahpasa Medical Faculty, Istanbul University-Cerrahpasa, Istanbul, Turkey

**Keywords:** *Magnusiomyces capitatus*, *Magnusiomyces clavatus*, *Saprochaete*, Antifungal susceptibility, Virulence factors, Diseases, Microbiology

## Abstract

Invasive infections due to *Magnusiomyces*/*Saprochaete* species are an emerging problem in immunocompromised patients and are often underrecognized because of misidentification and intrinsic resistance to some antifungals. This multicenter study investigated the species distribution, antifungal susceptibility patterns, and key virulence traits of clinical isolates from Türkiye. A total of 133 clinical isolates collected between 2010 and 2024 from 18 hospitals in 10 cities were identified by MALDI-TOF MS and ITS/LSU sequencing. MICs of amphotericin B, fluconazole, voriconazole, itraconazole, posaconazole, and flucytosine were determined using the EUCAST broth microdilution method. Biofilm formation and esterase, caseinase, secreted aspartyl proteinase, phospholipase, and hemolysin activities were assessed phenotypically. Sequencing identified 107 isolates (80.4%) as *Magnusiomyces capitatus* and 26 (19.6%) as *Magnusiomyces clavatus*, MALDI-TOF MS identified 106 (79.7%) as *M. capitatus* and 27 isolates (20.3%) as *M. clavatus*. There was 99.2% agreement between MALDI-TOF MS and sequencing results. Voriconazole, amphotericin B, and posaconazole showed the lowest MICs, whereas fluconazole displayed wide MIC ranges and limited activity. Overall, 97.7% of isolates were strong biofilm producers, with significantly higher biofilm production in *M. capitatus*. In contrast, *M. clavatus* showed higher caseinase and esterase activity. This study provides the most extensive multicenter dataset on *Magnusiomyces*/*Saprochaete* in Türkiye and underscores their considerable pathogenic potential through strong biofilm formation and tissue-degrading enzyme activities. Accurate species-level identification using MALDI-TOF MS supported by molecular methods is essential, and limited fluconazole activity suggests that voriconazole and amphotericin B should be prioritized in species-guided treatment strategies.

## Introduction

Invasive fungal infections (IFIs) are a major cause of morbidity and mortality in immunocompromised and critically ill patients. The presence of predisposing factors such as organ and tissue transplantation, surgical procedures, broad-spectrum antibiotics and immunosuppressive therapy, central venous catheters, and mechanical ventilation has contributed to the increasing incidence of opportunistic infections^1,2^.

It is estimated that IFIs are responsible for approximately 1.5 million deaths worldwide each year, with more than 80% of these deaths attributed to infections caused by *Candida* (particularly *C. albicans* and resistant strains), *Aspergillus* (*A. fumigatus*), and *Cryptococcus* (*C. neoformans)*^3,4^. Over the past decade, an increase in IFI cases due to yeasts such as *Magnusiomyces*/*Saprochaete* and *Trichosporon* species has also been reported, with a clinical spectrum ranging from superficial infections to catheter-related fungemia, peritonitis, and disseminated haematogenous infections^5^. In immunocompromised patients, the clinical presentation of fungal infections is often nonspecific and complex, making early diagnosis challenging and contributing to poor clinical outcomes^6^. Therefore, accurate identification of fungal isolates recovered from clinical specimens is critical for the selection of appropriate antifungal therapy and for preventing the spread of these pathogens in susceptible patient populations^7^.

*Magnusiomyces/Saprochaete* species are yeast-like fungi belonging to the family *Dipodascaceae* within the phylum *Ascomycota*. Isolates of this genus are widely distributed in nature, particularly in soil, water, air, plants, and grains^5,8^. They may also be detected as part of the human mycobiota on the skin and on the mucosa of the respiratory and gastrointestinal tracts. These yeasts are non-fermentative and urease-negative^8,9^. Based on molecular phylogenetic studies and nomenclatural priorities, species previously classified under the asexual genus *Saprochaete* have been reclassified into *Magnusiomyces*^10^. Currently, the genus comprises 18 species, among which *Magnusiomyces capitatus* and *M. clavatus* are the most frequently encountered pathogens^8,10^.

*Magnusiomyces clavatus* is morphologically and genetically closely related to *M. capitatus*. Both species produce white, indented colonies composed of true hyphae and arthroconidia^11^. However, their phenotypic similarities often lead to misidentification in routine diagnostic settings^11,12^. Treatment of these infections remains challenging due to limited antifungal options, intrinsic resistance to echinocandins, limited in vitro activity of fluconazole, and high mortality rates^12^.

*Magnusiomyces* species remain classified as rare pathogens; nevertheless, in recent years, an increasing frequency of infections has been reported, particularly in immunocompromised patients and in association with sporadic cases and nosocomial outbreaks^8–14^. In Türkiye, only a limited number of case reports on this pathogen have been published, and to date, a multicenter study focusing on parameters such as epidemiology, virulence traits, and antifungal susceptibility has not yet been conducted^15–23^. Thus, consolidating data on *Magnusiomyces* isolates from multiple centres is crucial for advancing our understanding of these rare but emerging pathogens and for guiding future clinical and experimental research.

Accordingly, the present study has been designed as the first multicenter investigation in Türkiye focusing on opportunistic *Magnusiomyces* species. This multicentre study aims to accurately identify isolates previously characterised by conventional methods to the species level using phenotypic and genotypic techniques, and to determine their antifungal susceptibility profiles and virulence properties.

## Methods

### Study design

This study was conducted in accordance with the Declaration of Helsinki and relevant national regulations. Ethical approval was obtained from the Ethics Committee of Nigde Omer Halisdemir University, Nigde, Türkiye (Approval No: 2022/92, Date: 14.09.2022). The requirement for informed consent was waived by the Ethics Committee of Nigde Omer Halisdemir University due to the retrospective nature of the study and the use of anonymized clinical isolates.

A total of 133 *Magnusiomyces* isolates preserved in the culture collections of medical microbiology laboratories were included in this study. These isolates were obtained between 2010 and 2024 from 18 hospitals located in 10 provinces across Türkiye. The isolates were transported to the reference laboratory under cold-chain conditions and stored at − 80 °C in a medium containing 15% glycerol until analysis.

### Identification of isolates

Isolates previously identified as *Saprochaete* spp., *S. clavata*, or *S. capitata* (synonyms: *Geotrichum clavatum*, *G. capitatum*, *Blastoschizomyces capitatus*,* Magnusiomyces capitatus*, *Magnusiomyces clavatus*) in the culture collections of participating centers using phenotypic methods (conventional techniques including colony and microscopic morphology, and carbohydrate assimilation tests) were re-identified to species level by matrix-assisted laser desorption ionization-time of flight mass spectrometry (MALDI-TOF MS) and sequencing analyses.

### MALDI-TOF MS analysis

Species identification of the isolates was performed using a MALDI-TOF MS system (Bruker Biotyper, database version 3.1, USA). Each isolate was subcultured on Sabouraud Dextrose Agar (SDA; Sigma-Aldrich, St. Louis, MO, USA) and incubated at 37 °C for 24–48 h to obtain isolated colonies. Isolated colonies were carefully selected with a sterile loop, placed onto a slide well, and treated with 1 µL of 70% formic acid. After air-drying, 1 µL of matrix solution (α-cyano-4-hydroxycinnamic acid, CHCA) was applied and allowed to dry at room temperature. Subsequently, the prepared plate was loaded into the MALDI-TOF MS instrument for spectral analysis^24^. According to the manufacturer’s criteria, scores of ≥ 2.0 indicated reliable species-level identification, scores between 1.7 and 2.0 indicated genus-level identification, while scores of < 1.7 indicated no reliable identification.

### DNA sequencing analysis

Genomic DNA was extracted from the isolates using the GeneAll Exgene™ Cell SV mini kit (GeneAll Biotechnology Co., Ltd., Korea). DNA concentration and purity (A260/280 ratio) were determined using a microplate spectrophotometer (Epoch 2, BioTek Instruments, USA). Samples were then stored at − 20 °C until further analysis. The internal transcribed spacer (ITS) region was amplified using the universal fungal primers ITS1 and ITS4. In addition, the large subunit (LSU) ribosomal gene region was amplified with the NL-1 and NL-4 primers. PCR products were enzymatically purified with ExoSap-IT (USB Corporation, Cleveland, OH, USA) before sequencing, which was performed using a modified protocol of the BigDye Terminator v3.1 Cycle Sequencing Kit (Applied Biosystems). Sequencing reaction products were purified using Sephadex G-50 (Sigma-Aldrich, St. Louis, MO, USA) and analyzed on an ABI PRISM 310 Genetic Analyzer (Applied Biosystems).

The sequences obtained in this study were aligned and compared with reference strains from GenBank, including *M. clavatus* CBS 425.71 (KF984489) and *M. capitatus* CBS 162.80 (KF984490). Comparative genomic analysis was performed to confirm species identification, and the newly generated sequences were deposited in GenBank. All ITS region sequences were aligned using Geneious Prime, and a phylogenetic tree was constructed using the Neighbor-Joining algorithm in MEGA 11. The resulting phylogenetic tree was visualized and annotated using the Interactive Tree of Life (iTOL) tool.

### In vitro antifungal susceptibility tests

The antifungal susceptibility of the isolates to amphotericin B (AMB), fluconazole (FLU), voriconazole (VOR), itraconazole (ITR), posaconazole (POS), and 5-flucytosine (5-FC) was determined according to the EUCAST (European Committee on Antimicrobial Susceptibility Testing) reference microdilution method (E.Def 7.4)^25^. Antifungal drugs were prepared in RPMI 1640 medium (Thermo Fisher Scientific, Waltham, MA, USA) supplemented with 2% glucose, and twofold serial dilutions were made in sterile 96-well microtiter plates. Each well was inoculated with 100 µL of a yeast suspension to achieve a final inoculum density of 0.5–2.5 × 10^5^ CFU/mL as described previously^23^. The plates were incubated at 35 ± 2 °C for 24 ± 2 h, after which the optical density was measured at 530 nm. *Candida parapsilosis* ATCC 20,019 and *Candida krusei (P. kudriavzevii*) ATCC 6258 reference strains were used for quality control in each test. Minimum inhibitory concentrations (MICs) were defined as the lowest concentrations that resulted in 50% growth inhibition for azoles and flucytosine, and complete growth inhibition for amphotericin B.

### Investigation of in vitro virulence factors

The enzymatic and virulence-related activities of *Magnusiomyces* isolates, including hemolytic, phospholipase, proteinase, caseinase, and esterase activities, were evaluated using established plate-based methods.

For all enzymatic assays, a yeast suspension was prepared by subculturing isolates on Sabouraud Dextrose Agar (SDA) at 37 °C for 18–24 h. Cells were harvested, washed twice with sterile physiological saline, and adjusted to a final concentration of 1 × 10^8^ cells/mL. 10 µL of yeast suspension was spot inoculated onto the specific agar media. Each isolate was tested in duplicate, and the mean value was reported^26^.

*Haemolytic activity* According to Luo et al.^27^, hemolytic activity was assessed on 7% enriched sheep blood Sabouraud Dextrose Agar. After incubation at 37 °C in a 5% CO₂ atmosphere for 48 h, the presence of a translucent halo around the colony was recorded as a positive hemolytic reaction. Haemolytic index (HI) values were calculated from colony and halo diameters.

*Phospholipase activity* Phospholipase production was determined using the egg yolk agar (EYA) method described by Price et al.^28^, with the plates being incubated at 37 °C for up to 5 days. The enzyme activity was then evaluated based on the phospholipase zone (Pz) value, which was interpreted as follows: Pz = 1.0 (no activity); 0.89–0.99 (weak); 0.70–0.89 (moderate); and < 0.70 (strong activity).

*Proteinase activity* Proteinase activity was tested using bovine serum albumin (BSA) agar plates incubated at 37 °C for 5 days^29^. After fixation with 20% trichloroacetic acid, a clear zone around colonies indicated proteinase activity. Proteolytic activity was quantified by calculating the proteinase value (Pz), defined as the ratio of the colony diameter to the total diameter of the colony plus hydrolysis zone.

*Caseinase activity* Casein hydrolysis was evaluated on skim milk agar. After incubation at 37 °C for 2–3 days, a clear zone around colonies indicated caseinase activity^30^.

*Esterase activity* Esterase activity was determined using the Tween 80 agar as described by Rudek.^31^. Plates were incubated at 30 °C for 3 days, with an opaque halo around the colony indicating positive activity.

*Biofilm formation* The biofilm-forming ability of *Magnusiomyces* isolates was assessed using a modified microplate biofilm assay based on the method of Thein and Salari^32,33^. Isolates grown on SDA were suspended in RPMI-1640 medium to a concentration of 10⁶ CFU/mL, incubated overnight, and the cell density was then adjusted to 10⁷ cells/mL. For biofilm development, standardized cell suspensions were dispensed in triplicate into 96-well microtiter plates. The plates were incubated for 2 h to allow initial adhesion, and then the medium was gently replaced. Incubation was continued at 37 °C with shaking for 24 h. Biofilm biomass was then quantified using the MTT reduction assay. Following washing with PBS to remove non-adherent cells, 20 µL of MTT solution (5 mg/mL; Sigma-Aldrich, St. Louis, MO, USA) was added to each biofilm and control well, and plates were incubated at 37 °C in the dark for 4 h. Supernatants were discarded, 100 µL of dimethyl sulfoxide (DMSO; Merck, Darmstadt, Germany) was added to each well and incubated at room temperature for 10 min, and the optical density (OD) was measured at 570 nm using a microplate reader (Epoch 2, BioTek Instruments, USA). The biofilm-forming capacity was assessed according to the classification proposed by Stepanović et al.^34^. Accordingly, the isolates were classified as non-biofilm producers (OD ≤ ODc), weak biofilm producers (ODc < OD ≤ 2 × ODc), moderate biofilm producers (2 × ODc < OD ≤ 4 × ODc), and strong biofilm producers (4 × ODc < OD). To ensure reproducibility and reliability of the results, all experiments were performed in triplicate.

### Statistical analysis

All analyses were performed using IBM SPSS Statistics (version 18; IBM Corp., Armonk, NY, USA). Demographic, clinical, antifungal susceptibility, and virulence data were summarised using descriptive statistics. A p value < 0.05 was considered statistically significant.

## Results

### Types of clinical samples and patient profiles

This study retrospectively analyzed the demographic and clinical characteristics of patients from whom 133 *Magnusiomyces* isolates were obtained. The mean age of patients was 58.8 years (median 61; range 2–91), and infections were predominantly observed in individuals aged 60 years and older, although rare cases were also reported in pediatric and young adult populations. The majority of isolates originated from elderly male patients. A male predominance was noted, with 72.2% (*n* = 96) of isolates from male patients and 27.8% (*n* = 37) from female patients (Table 1).

The isolates were most frequently recovered from urine (28.6%, *n* = 38), sputum (24.8%, *n* = 33), and endotracheal aspirates (16.5%, *n* = 22). Blood samples also accounted for 16.5% (*n* = 22) of the isolates. Bronchoalveolar lavage (BAL) and catheter samples constituted 5.3% (*n* = 7) and 4.5% (*n* = 6), respectively. Less common sources included pus (*n* = 2, 1.5%), cerebrospinal fluid (CSF) (*n* = 1, 0.8%), biopsy (*n* = 1, 0.8%), and bronchial lavage (*n* = 1, 0.8%) (Table 1).

**Table 1:** Age and gender of patients, and type of clinical samples.

Demographic and basic clinical data	
Characteristics	Total (*n* = 133)
Age, Median (Range)	61 (2–91) years
*Sex*,* n (%)*	
Female	37 (27.8%)
Male	96 (72.2%)
*Clinical samples*	
Urine	38 (28.6%)
Sputum	33 (24.8%)
Blood	22 (16.5%)
Endotracheal aspirate	22 (16.5%)
Bronchoalveolar Lavage (BAL)	7 (5.3%)
Catheter	6 (4.5%)
Pus	2 (1.5%)
Cerebrospinal fluid (CSF)	1 (0.8%)
Biopsy	1 (0.8%)
Bronchial lavage	1 (0.8%)

### Distribution of isolates

**Fig. 1:**
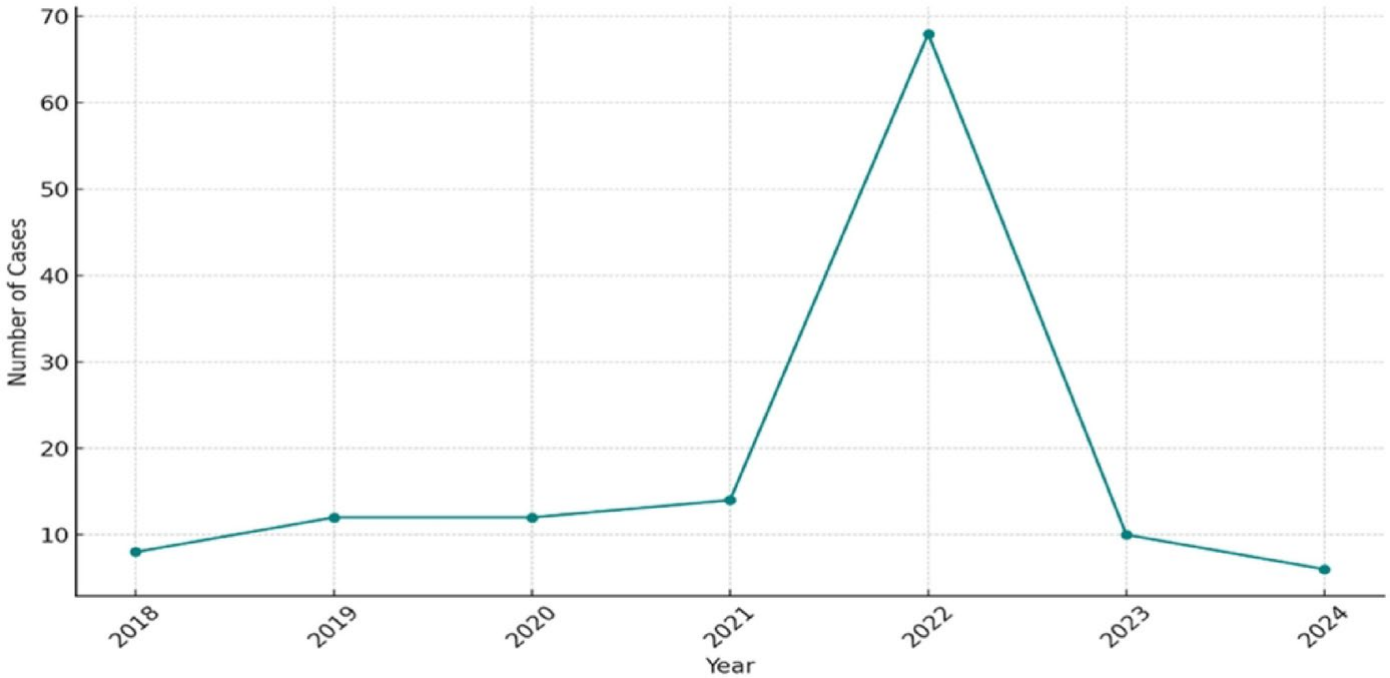
Temporal distribution of *Magnusiomyces* isolates between 2018 and 2024 (data from 2010–2017 not shown due to sporadic isolation and low annual case numbers.

Between 2010 and 2024, the distribution of isolates exhibited a heterogeneous pattern. While sporadic cases were reported between 2010 and 2013, a gradual increase was observed starting in 2018. This trend culminated in a marked peak in 2022, accounting for 68 isolates (51.1% of the total cohort), followed by a decline in case numbers in 2023 and 2024 (Fig. 1).

### Identification of isolates

**Fig. 2:**
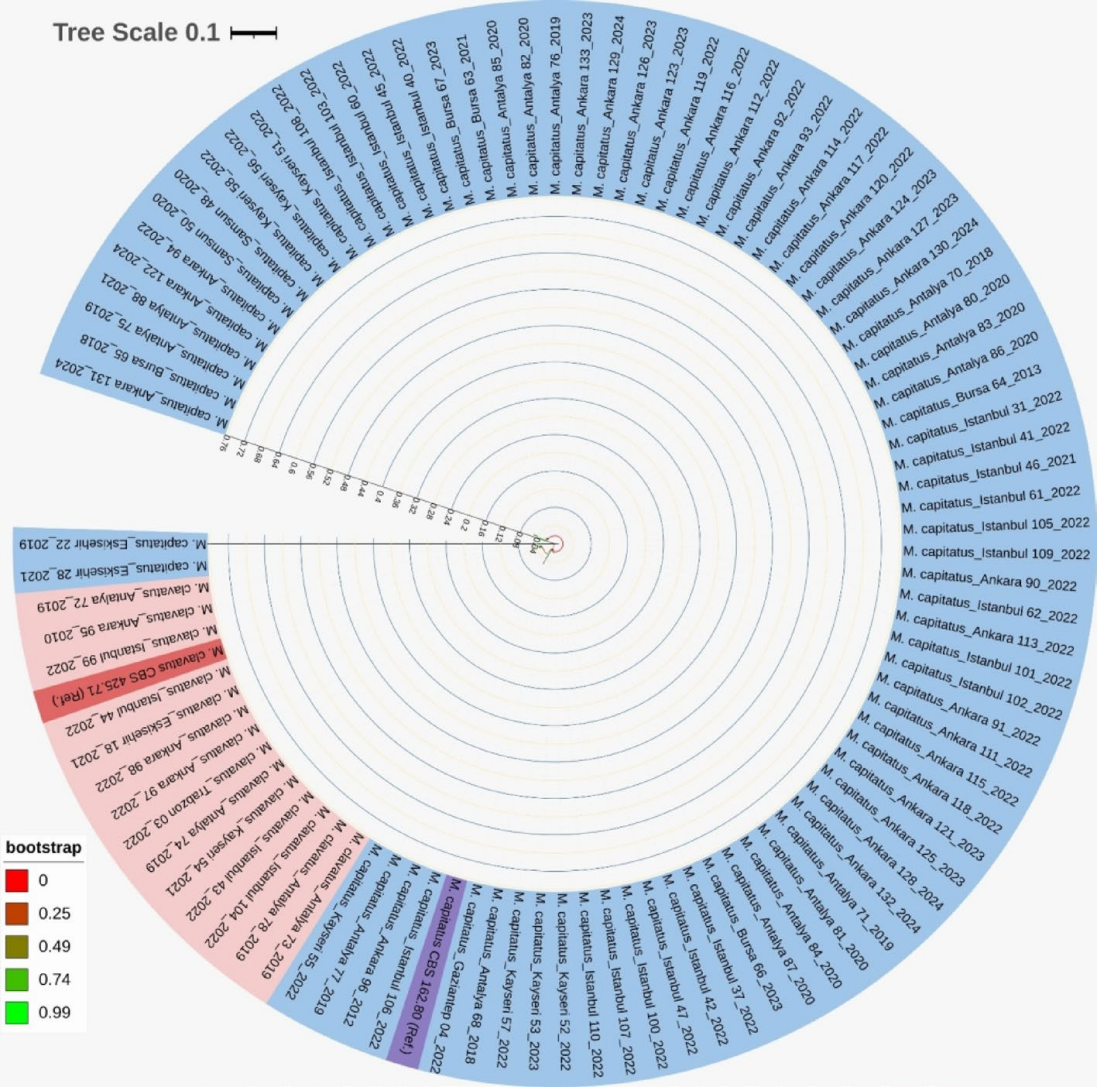
The phylogenetic tree of *Magnusiomyces capitatus* and *Magnusiomyces clavatus* isolates was constructed based on the ITS region. Clinical isolates collected from different regions of Türkiye are shown, with branch colors representing the sampling provinces. The tree illustrates the geographic distribution and clustering patterns of the isolates, allowing comparison of phylogenetic relationships in relation to their regional origins. Reference strains (*M. capitatus* CBS 162.80 and *M. clavatus* CBS 425.71) are also included for comparison. The phylogeny was reconstructed using the Neighbor-Joining algorithm.

In this multicentre study, 133 *Magnusiomyces* isolates obtained from clinical samples were analysed using MALDI-TOF MS and Sanger sequencing for species-level identification. For Sanger sequencing, the ITS gene region was sufficient for identifying 96 isolates, while the LSU gene region was sequenced for the discriminative identification of the remaining 37 isolates. Sanger sequencing identified 26 isolates (19.6%) as *M. clavatus* and 107 isolates (80.4%) as *M. capitatus*, while MALDI-TOF MS identified 27 isolates (20.3%) as *M. clavatus* and 106 isolates (79.7%) as *M. capitatus*. A very high concordance between the two methods was observed for 132 isolates (99.2%), with only one isolate (0.8%, Isolate 18) showing discordance. The discordant isolate, classified as *M. capitatus* by MALDI-TOF MS, was ultimately assigned as *M. clavatus* based on sequencing. The ITS/LSU sequences were indistinguishable from or very similar to those of other *M. clavatus* isolates in our cohort. The obtained sequence data were deposited in the GenBank database under accession numbers PV273725–PV273820, as well as MK834539.1, LC497444.1, and MK834530.1. Phylogenetic analysis based on ITS sequences revealed that isolates from different hospitals and collection years were distributed throughout the tree without forming distinct clusters. No significant correlation was observed between the genetic diversity of the isolates and their geographic origin or isolation date, suggesting a heterogeneous population structure across the participating centers (Fig. 2).

### Antifungal susceptibility testing

The MIC values determined for 133 *Magnusiomyces* isolates were summarized in Table 2. Since no clinical breakpoints are available for any of the antifungal agents tested against *Magnusiomyces*, the isolates were not categorized as susceptible/resistant. The MIC ranges were broad in general for all drugs and both species. The degree of *in vitro* activities of the drugs was ranked depending on the MIC values. The ranking order against *M. capitatus* strains was VOR ~ AMB~POS > ITR~5-FC > FLU. For *M*. *clavatus* isolates, on the other hand, the ranking order was as VOR ~ AMB~POS ~ ITR>5-FC > FLU. Fluconazole MICs ranged from 0.125 to 64 mg/L for *M. capitatus* and from 2 to 64 mg/L for *M. clavatus*, indicating variable activity among isolates but generally higher MICs compared with the other antifungals.

**Table 2:** Antifungal susceptibility test results of *Magnusiomyces* isolates.

Species (*n*)	Antifungal	MIC₅₀ (mg/L)	MIC₉₀ (mg/L)	MIC range (mg/L)
*M. capitatus* (*n* = 107)	AMB	1	2	0.125–4
	FLU	8	32	0.125–64
	VOR	0.125	2	0.008–4
	ITR	0.125	8	0.008–>8
	POS	0.125	2	0.008–>4
	5-FC	0.125	8	0.125–32
*M. clavatus* (*n* = 26)	AMB	1	2	0.5–2
	FLU	16	32	2–64
	VOR	0.25	2	0.016–2
	ITR	0.5	2	0.03–8
	POS	0.5	2	0.06–2
	5-FC	0.25	16	0.125–32

*AMB* amphotericin B, *FLU* fluconazole, *VOR* voriconazole, *ITR* Itraconazole, *POS* posaconazole, *5-FC* flucytosine.

### Virulence factors of isolates

The distribution of virulence factors among *M. capitatus* and *M. clavatus* isolates is summarized in Table 3, which presents detailed results of the virulence assays. In this study, the most common virulence trait detected among *M. capitatus* isolates was the ability to form biofilms. Notably, 99.0% of isolates demonstrated strong biofilm-forming ability, while only 0.9% showed moderate biofilm formation. After biofilm formation, esterase activity was the second most common virulence factor detected in 52.3% of isolates. Caseinase activity was observed in 27.1% of isolates, along with 8.4% exhibiting secreted aspartyl proteinase (SAP) activity, 4.7% showing hemolytic activity, and only 0.93% displaying phospholipase activity.

The majority (92.3%) of *M. clavatus* isolates displayed a strong biofilm-forming ability, while only two isolates (7.7%) showed weak biofilm-forming ability. Esterase activity was present in 61.5% of the isolates, whereas proteinase activity was relatively uncommon (7.7%). None of the isolates demonstrated phospholipase activity. Caseinase activity was detected in most isolates (80.8%), with 30.8% (eight isolates) exhibiting very strong activity. No haemolytic activity was observed in any isolate.

Despite the high overall rates of biofilm formation in both species, *M. capitatus* isolates exhibited a statistically significantly higher proportion of strong biofilm producers compared to *M. clavatus* (99.0% vs. 92.3%, *p* = 0.04). Similarly, caseinase activity was significantly more prevalent in *M. clavatus* isolates than in *M. capitatus* (84.6% vs. 27.1%, *p* = 0.001).

**Table 3:** Distribution of virulence factors among *Magnusiomyces* species (*n* = 133).

Virulence factor	*M. capitatus* (*n* = 107)	*M. clavatus* (*n* = 26)	Total (*n* = 133)	*p* value
Proteinase	9 (8.4%)	2 (7.7%)	11 (8.3%)	0.693
Phospholipase	1 (0.9%)	0 (0%)	1 (0.8%)	1.000
Esterase	56 (52.3%)	17 (65.4%)	73 (54.9%)	0.343
Hemolytic activity	5 (4.7%)	0 (0%)	5 (3.8%)	0.448
Caseinase	29 (27.1%)	22 (84.6%)	51 (38.3%)	0.001
*Biofilm formation*				
Strong	106 (99.0%)	24 (92.3%)	130 (97.7%)	0.04
Moderate	1 (0.9%)	0 (0%)	1 (0.8%)	
Weak	0 (0%)	2 (7.7%)	2 (1.5%)	0.095

## Discussion

In recent years, the incidence of infections caused by *Magnusiomyces* species has increased, particularly in patients with hematological malignancies or immunosuppressive conditions^35^. However, data on their diagnosis, antifungal susceptibility, and virulence properties are still limited. To the best of our knowledge, this study represents the most comprehensive investigation to date, including species-level identification, antifungal susceptibility testing, and virulence profiling of 133 isolates obtained from 18 hospitals in 10 provinces of Türkiye.

Most isolates were obtained from elderly individuals (47% were ≥ 60 years) and male patients (72.2%), suggesting a potential association between these infections and advanced age or male gender. This finding is consistent with previous reports linking hematological malignancies with increased susceptibility to these infections^17,22^. Koç et al. reported that all three cases of fungemia they described occurred in male patients, which is consistent with our findings^16^. However, the presence of pediatric cases in our cohort indicates that these infections are not limited to older populations. Soler-Simón et al. reported a case of disseminated *M. capitatus* infection in a child with Burkitt lymphoma, while Özkaya-Parlakay et al. described a pediatric case of *G. capitatum* fungemia associated with galactomannan positivity^15,36^. Erman et al. documented an invasive *S. capitata* infection in a patient with congenital CARD9 deficiency, demonstrating that individuals with both acquired and innate immunodeficiencies may be predisposed to these infections.

Urine was the most common source of *Magnusiomyces* isolation (28.6%), suggesting that these organisms can cause both localized and systemic infections. This is supported by a case reported by Hazirolan et al.^20^, in which a urinary tract infection occurred during anidulafungin therapy, illustrating the broad spectrum of clinical manifestations associated with these fungi. Furthermore, the frequent isolation of *Magnusiomyces* species from respiratory specimens and blood cultures indicates a high potential for systemic dissemination. These observations are consistent with previous reports, including *S. clavata*-associated sepsis described by Kangül et al.^22^, and *S. capitata*-induced fungaemia and septic arthritis documented by Parkan et al.^19^. Notably, the isolation of *M. capitatus* from the oral mucosa, as described by Ghojoghi et al.^37^, highlights the diagnostic challenges in differentiating colonization from true infection.

The temporal distribution of isolates in our study exhibited a significant peak in 2022 (Fig. 1). This specific increase coincided with the post-acute phase of the COVID-19 pandemic, a period characterized by high ICU occupancy rates, prolonged hospitalizations, and the widespread use of broad-spectrum antibiotics and corticosteroids. These factors are well-established risk factors for opportunistic fungal infections. Although the retrospective nature of our investigation and the lack of national denominator data limit the establishment of a definitive causal association, the observed increase in 2022 may reflect the cumulative impact of pandemic-related healthcare challenges. The subsequent decline in incidence rates in 2023 and 2024 suggests a potential normalization, possibly attributable to the reimplementation of standard infection control protocols and diagnostic routines.

Regarding diagnostic strategies, both MALDI-TOF MS and Sanger sequencing were employed in this study to achieve species-level identification. The observed concordance rate of 99.24% between these methods highlights the value of MALDI-TOF MS as a rapid and reliable diagnostic tool. Nevertheless, the minor discrepancies detected in a subset of isolates likely reflect limitations of currently available spectral libraries or analytical algorithms. For closely related species, confirmatory testing with molecular methods such as Sanger sequencing therefore remains essential. Our findings thus support the use of MALDI-TOF MS for routine identification while also underscoring the need for supplementary molecular validation in diagnostically challenging or ambiguous cases. The ongoing expansion and refinement of spectral databases are expected to further improve the diagnostic accuracy of MALDI-TOF MS in the future. Molecular characterisation in our cohort showed that 80.4% of isolates were identified as *M. capitatus* and 19.6% as *M. clavatus*, closely consistent with the distribution earlier reported from Türkiye by Kaşaltı et al.^23^.

The susceptibility profiles observed in this study align with those reported in previous studies, such as that by Kraft et al., which demonstrated relatively low MICs, suggesting susceptibility to posaconazole and amphotericin B among Brazilian isolates^38^. On the other hand, the elevated MIC values for fluconazole against both species (and for itraconazole, particularly in comparison to voriconazole and posaconazole, against *S. capitata*) observed in our study mirror the MIC distributions previously reported by Koç et al.^18^ and Pamidimukkala et al.^39^. These in vitro susceptibility profiles were further highlighted in the case of a post-liver transplant patient with a mycotic aneurysm reported by Gilbert et al.^40^.

Among the virulence factors evaluated, the predominance of biofilm-forming capacity among the isolates is of clinical relevance, given its well-established role in enhancing antifungal resistance. *Magnusiomyces* isolates were reported as strong biofilm producers previously^23^. Kraft et al. reported that biofilm-positive isolates are associated with higher rates of therapeutic failure. In addition, the *M. clavatus* isolates in our study exhibited pronounced esterase and caseinase activities, indicating a higher potential for tissue invasion. This observation is consistent with the invasive disease and unfavorable clinical outcomes described in *M. clavatus* infections by Kangül et al.^22^ and Sprute et al.^41^.

The significantly higher caseinase activity in *M. clavatus* suggests a potent proteolytic capability, which may facilitate deep tissue invasion and dissemination from non-sterile sites, such as sputum or urine. In contrast, the universal strong biofilm production in *M. capitatus* aligns with its frequent isolation from indwelling devices such as catheters and its persistence in hospital environments, potentially contributing to nosocomial clusters.

Integrating the antifungal susceptibility profiles with virulence characteristics offers crucial insights for therapeutic strategies. The convergence of limited fluconazole activity and strong biofilm-forming capacity in both *M. capitatus* and *M. clavatus* presents a dual challenge for clinical management. Since biofilm-associated cells are inherently more resistant to antifungal agents, the standard empiric use of fluconazole in high-risk patients may be ineffective against these emerging pathogens. Consequently, in patients with hematological malignancies or indwelling devices presenting with yeast infections, our findings support the early consideration of alternative agents such as voriconazole or amphotericin B, which demonstrated superior *in vitro* activity in our cohort.

Our results aligned with prior case reports and small series; however, the multicenter design and large sample size of our study enabled a more rigorous assessment of phenotypic traits and antifungal susceptibility patterns. By incorporating detailed virulence factors, including biofilm formation and enzymatic activity, together with patient demographics, we gained valuable insights into the pathogenicity of these uncommon fungi. Based on data from 18 centers across Türkiye, this study represents one of the most comprehensive regional analyses of *Magnusiomyces* infections to date.

However, the study’s retrospective nature and the complexities of multicenter data collection limited our access to detailed, long-term clinical follow-up for some cases. Consequently, our analyses focused on core demographic variables (age, sex) and specimen types that were consistently available for all participants. Key variables, including underlying immunodeficiencies, characteristics of neutropenia, definitions of infection, treatment strategies, and outcomes, were excluded from the analysis due to incomplete recording. In addition, the lack of detailed clinical information regarding malignancy types, neutropenia duration, and concurrent infections limited the assessment of host-related risk factors. Although this limitation reduces our ability to correlate antifungal susceptibility findings with clinical outcomes, the study’s primary objective of microbiologically characterizing the isolates, including their antifungal susceptibility and *in vitro* virulence properties, has been robustly and reliably addressed using the available data.

## Conclusions

In conclusion, *Magnusiomyces* species display a wide clinical spectrum and varying MIC values of antifungal drugs in clinical use are observed against *Magnusiomyces* isolates. The higher in vitro activities of voriconazole, amphotericin B, and posaconazole against both *Magnusiomyces* species, as well as the limited activity of fluconazole, are noteworthy. Virulence attributes, particularly biofilm formation and tissue-degrading enzymatic activities, likely contribute significantly to their pathogenic potential. Accurate species identification remains critical for effective antifungal management. While MALDI-TOF MS offers rapid and practical identification, Sanger sequencing continues to be indispensable, especially in ambiguous or clinically significant cases. Improvement in diagnostic and treatment modalities will depend on future studies leveraging whole-genome sequencing, proteomic analyses, and well-designed clinical investigations.

## Data Availability

The DNA sequence datasets generated in this study and supporting the findings are openly available in NCBI GenBank at “https://www.ncbi.nlm.nih.gov/nuccore/?term=PV273725:PV273820[accn]” under accession numbers PV273725–PV273820.

## Abbreviations

AMB: Amphotericin B
BAL: Bronchoalveolar Lavage
CFU: Colony forming unit
CSF: Cerebrospinal fluid
DMSO: Dimethyl sulfoxide
DNA: Deoxyribonucleic acid
EUCAST: European Committee on Antimicrobial Susceptibility Testing
FLU: Fluconazole
ICU: Intensive care unit
IFIs: Invasive fungal infections
ITS: Internal transcribed spacer
ITR: Itraconazole
LSU: Large subunit (ribosomal DNA)
MALDI-TOF MS: Matrix-assisted laser desorption ionisation-time-of-flight mass spectrometry
MIC: Minimum inhibitory concentration
ML: Maximum likelihood
MTT: 3-(4,5-Dimethylthiazol-2-yl)-2,5-diphenyltetrazolium bromide (assay)
OD: Optical density
PBS: Phosphate-buffered saline
PCR: Polymerase chain reaction
POS: Posaconazole
RPMI: Roswell Park Memorial Institute (medium)
SAP: Secreted aspartyl proteinase
SDA: Sabouraud dextrose agar
VOR: Voriconazole
5-FC: 5-Flucytosine
